# Bone Cement Leakage‐Induced Pulmonary Embolism: A Case Report

**DOI:** 10.1002/ccr3.72959

**Published:** 2026-06-23

**Authors:** Yang Xv, Ye Bo

**Affiliations:** ^1^ Hebei North University Heibei China; ^2^ Air Force Specialized Medical Center Beijing China

**Keywords:** bone cement embolism, bone cement leakage, case report, kyphoplasty, pulmonary embolism, vertebroplasty

## Abstract

Pulmonary cement embolism (PCE) is a rare but potentially fatal complication of bone cement augmentation procedures. We report a 61‐year‐old woman who developed profound hypoxemia immediately after lumbar decompression, fusion, and bone cement augmentation. Postoperative imaging demonstrated pulmonary cement embolism, bilateral pulmonary infiltrates, and progressive cardiopulmonary deterioration. Despite intensive care, mechanical ventilation, anticoagulation, and vasoactive support, the patient developed acute respiratory distress syndrome (ARDS) and died on postoperative day 13. This case highlights the importance of preventing cement leakage, early postoperative imaging, and prompt recognition of pulmonary cement embolism.

## Key Clinical Message

A 61‐year‐old female patient died on postoperative day 13 following lumbar bone cement surgery. She experienced immediate, profound hypoxemia attributed to pulmonary cement embolism, and subsequently developed pneumonia, acute respiratory distress syndrome (ARDS), and pulmonary hypertension despite intensive therapeutic interventions.

## 1 Introduction

Bone cement augmentation is widely used for vertebral compression fractures due to its rapid pain relief and stabilizing effect, but it carries the risk of severe complications such as pulmonary cement embolism (PCE). Although uncommon, PCE can progress rapidly and become life‐threatening. This case demonstrates a fatal course following cement leakage, highlighting the importance of early detection and preventive strategies.

## 2 Case History and Examination

A 61‐year‐old woman presented on March 5, 2025, with worsening symptoms after seven months of failed conservative management for a traumatic lumbar compression fracture. Lumbar MRI revealed chronic fractures at T12 and L1 with endplate osteitis, acute/subacute compression fractures at L2 and L4 with bone marrow edema, and disc injuries at L3/4 and L4/5. Preoperative chest radiography showed clear lung fields and a normal cardiac silhouette, indicating preserved cardiopulmonary function.

## 3 Differential Diagnosis, Investigations and Treatment

**FIGURE 1:**
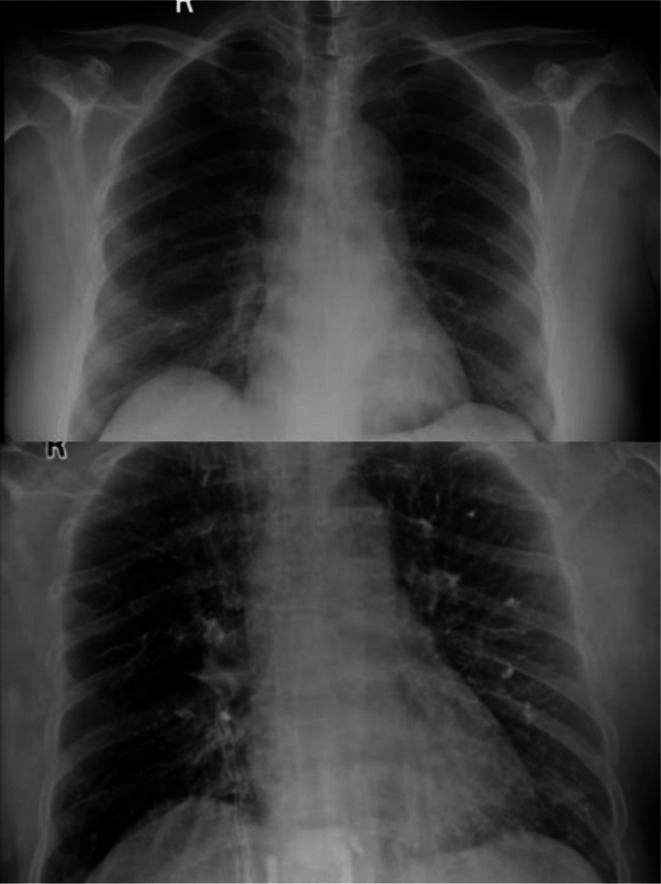
Preoperative and postoperative chest X‐rays.

**FIGURE 2:**
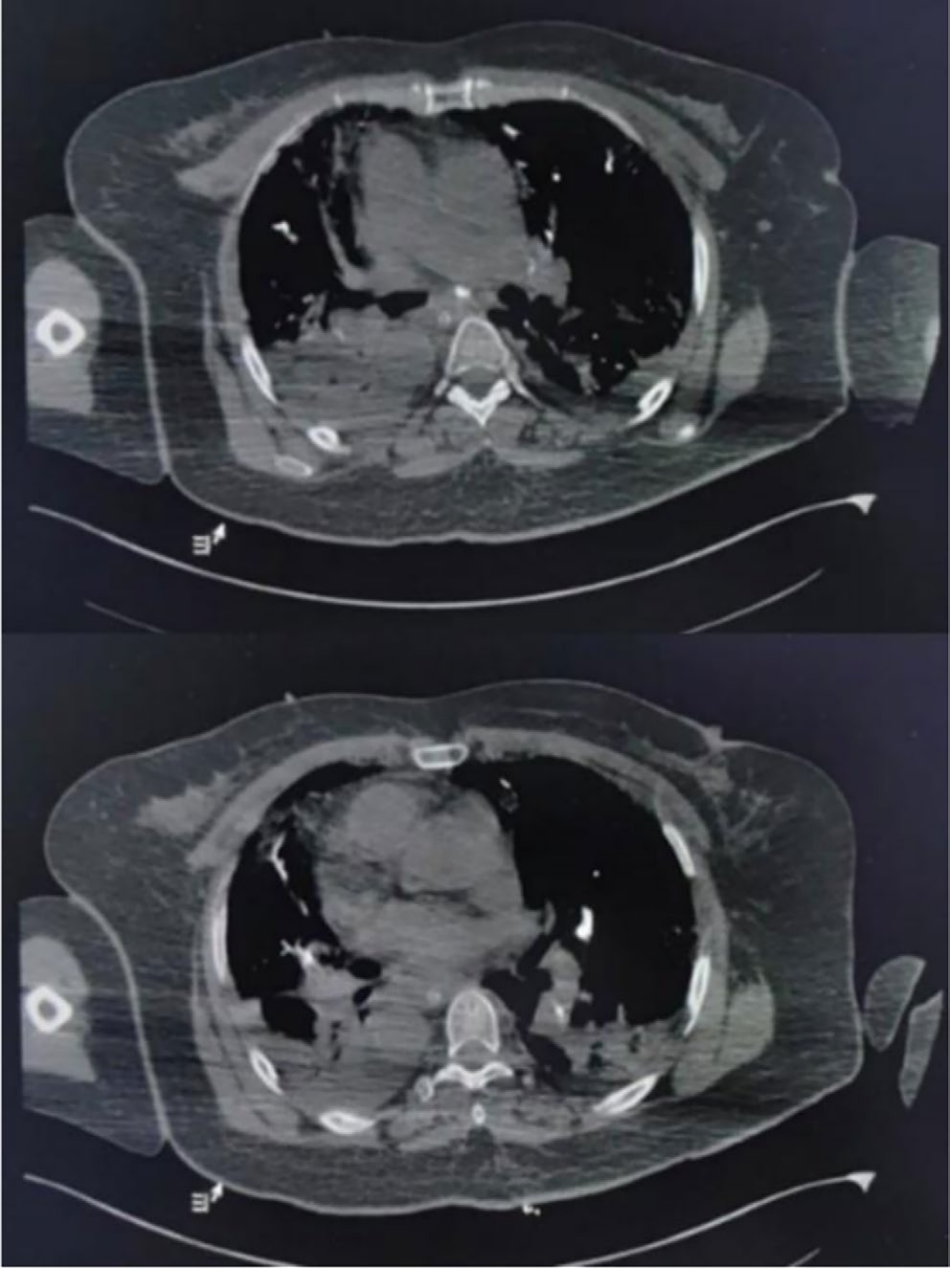
Postoperative chest CT.

**FIGURE 3:**
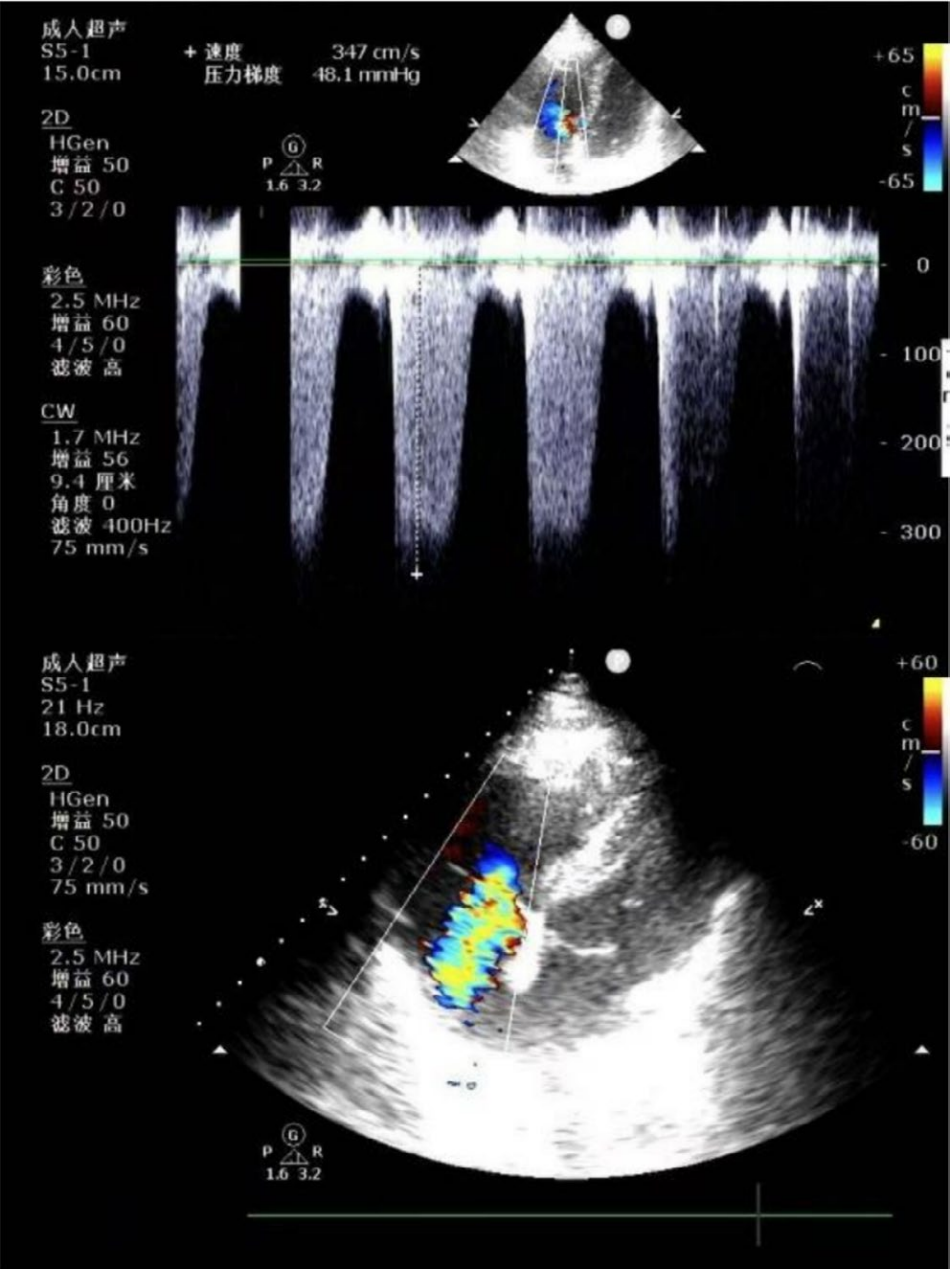
Preoperative and postoperative echocardiography.

On March 12, the patient underwent lumbar spinal canal decompression, interbody fusion with internal fixation, and bone cement augmentation under general anesthesia. Intraoperative blood loss was substantial (approximately 2000 mL), requiring transfusion of autologous blood, packed red blood cells, and plasma. A brief hypoxemic episode (SpO_2_ 88%) occurred but improved after intervention, and diphenhydramine was administered prophylactically for potential cement‐related allergic reactions. Severe hypoxemia (SpO_2_ 70%) occurred during postoperative repositioning, prompting immediate ICU transfer. In the ICU, she developed shock, persistent hyperthermia, hemodynamic instability, and progressive respiratory failure. Chest imaging (Figures 1 and 2) showed increased pulmonary vascular markings, dense linear opacities along pulmonary arteries, extensive bilateral ground‐glass opacities, and partial lower‐lobe atelectasis, consistent with pulmonary cement embolism, pneumonia, and atelectasis. Echocardiography demonstrated pulmonary hypertension and moderate‐to‐severe tricuspid regurgitation (Figure 3). Despite mechanical ventilation, anticoagulation, and vasoactive support, the patient died of ARDS secondary to cement embolism and pneumonia on March 25, 2025.

## 4 Conclusion and Results

The patient experienced rapid clinical deterioration following cement leakage, ultimately developing ARDS and fatal cardiopulmonary collapse despite aggressive treatment. This case underscores the potentially devastating consequences of pulmonary cement embolism and the importance of prompt recognition and comprehensive perioperative monitoring.

## 5 Discussion

Bone cement augmentation is an established treatment for lumbar vertebral compression fractures, offering immediate vertebral stabilization and pain relief, but it is also associated with complications such as adjacent vertebral fractures, cement leakage, infection, and vascular embolism [1]. Among these, cement leakage represents the most heterogeneous and potentially severe event. Intradiscal leakage may accelerate adjacent segment degeneration through metabolic disruption and altered load transmission, while vascular leakage—via valveless venous pathways including the extravertebral venous plexus or azygos vein—can result in pulmonary cement embolism (PCE) or even cardiac perforation; paraspinal leakage may also provoke neural irritation [2]. Reported PCE incidence ranges widely from 2.1%–26% in retrospective studies to 24%–28.6% in prospective studies, primarily due to differences in imaging vigilance [3, 4]. A multicenter study by Tamagawa et al. [5] involving 140 patients and 650 cement‐augmented screws identified low bone mineral density, cortical defects—especially posterior wall compromise—larger cement volumes, and low‐viscosity cement injection as independent risk factors for leakage. Although intravascular cement fragments may remain asymptomatic [6], even clinically silent PCE carries the potential for delayed cardiopulmonary deterioration [3, 7]. Chest CT, characterized by its ability to depict tubular or branching hyperdense opacities within pulmonary arteries, remains the diagnostic gold standard with markedly superior sensitivity to radiography [8]. Therefore, postoperative chest imaging is recommended regardless of symptom presence [4, 8].

Perioperative management should prioritize leakage prevention and early detection. Recommended strategies include using high‐viscosity cement with careful control of injection timing and volume [9], adopting venous filtration techniques to intercept potential embolic material intraoperatively [10], and strictly avoiding reinfusion of salvaged blood potentially contaminated with cement particles or monomers. Current protocols advise systematic evaluation for cement leakage within 24 h after surgery, including cardiopulmonary imaging, followed by long‐term surveillance at 1, 2, and 5 years to identify delayed complications [11]. For confirmed PCE, treatment depends on symptomatology and embolus location: symptomatic peripheral or asymptomatic central emboli typically warrant 3–6 months of anticoagulation (heparin‐to‐warfarin bridging or direct oral anticoagulants) [3, 4], whereas symptomatic central emboli with hemodynamic compromise may require surgical embolectomy [4, 7]. Asymptomatic retained intravascular or non‐embolic cement fragments may be managed conservatively with individualized follow‐up.

## Author Contributions

**Yang Xv:** writing – original draft, writing – review and editing. **Ye Bo:** resources, supervision, validation.

## Funding

The authors have nothing to report.

## Consent

Written informed consent for publication of this case report and accompanying images was obtained from the patient's legal representative, as the patient passed away in the ICU on March 25, 2025.

## Data Availability

Data sharing is not applicable to this article as no datasets were generated or analyzed during the current study.
